# No difference in failure between static, articulating, and prosthetic low-friction spacers for periprosthetic joint infection of total knee arthroplasty

**DOI:** 10.5194/jbji-10-243-2025

**Published:** 2025-07-30

**Authors:** Michael F. Shannon, Timothy Edwards, Timothy Maurer, Andrew J. Frear, Victoria R. Wong, Shaan Sadhwani, Clair Smith, Anthony Kamson, Brian Omslaer, Christian Cisneros, Andrew Gordon, Akeem Williams, Neel B. Shah, Kenneth L. Urish

**Affiliations:** 1 School of Medicine, University of Pittsburgh, Pittsburgh, PA 15213, USA; 2 Department of Orthopaedic Surgery, UPMC Central PA, Harrisburg, PA 17109, USA; 3 School of Health and Rehabilitation Sciences, University of Pittsburgh, Pittsburgh, PA 15213, USA; 4 Division of Infectious Disease, Department of Internal Medicine, University of Pittsburgh, Pittsburgh, PA 15213, USA; 5 Arthritis and Arthroplasty Design Group, The Bone and Joint Center, Magee-Womens Hospital of the University of Pittsburgh Medical Center, Pittsburgh, PA 15213, USA; 6 Department of Orthopaedic Surgery, University of Pittsburgh, Pittsburgh, PA 15213, USA; 7 Department of Bioengineering, University of Pittsburgh, Pittsburgh, PA 15213, USA; 8 Clinical and Translational Science Institute, University of Pittsburgh, Pittsburgh, PA 15213, USA; 9 Department of Biomedical Engineering, Carnegie Mellon University, Pittsburgh, PA 15213, USA

## Abstract

**Introduction**: Two-stage revision with an antibiotic spacer is the gold-standard treatment of prosthetic joint infection (PJI) for total knee arthroplasty (TKA). Multiple spacer designs exist, including static, articulated, and prosthetic low-friction (PALF) spacers. However, current literature is limited on variant superiority for infection eradication. This study aimed to compare outcomes of two-stage exchange for TKA PJI between patients with static cement, articulated cement, and PALF spacers. **Methods**: This retrospective study included 93 patients who underwent two-stage revision for PJI following primary TKA and received a static ( n=17 ), articulating ( n=54 ), or low-friction ( n=22 ) spacer. The primary outcome was failure at 2 years, defined as spacer retention, reoperation, or death. Secondary outcomes included reimplantation and discontinued antibiotics by 1 year, time to failure, duration of hospital stay, functional measures, and adverse events. Outcomes were compared between groups using hypothesis testing for continuous or categorical measures. **Results**: At 2 years, no significant difference in failure was seen for static (58.82 %), articulating (35.19 %), and PALF (22.73 %) spacers ( p=0.064 ). Articulating spacers demonstrated greater range of motion than static spacers at the final follow-up ( p=0.0 3). Static spacers were associated with a higher adverse-event frequency ( p=0.03 ). No other significant differences in outcomes were observed (all p>0.05 ). **Conclusions**: The three spacer variants demonstrated similar failure rates for two-stage revision of TKA PJI at 2 years. Static spacers may lead to adverse events more frequently compared to other designs, and a longer interstage duration for prosthetic spacers may reflect greater functionality.

## 1 Introduction

**Figure 1:**
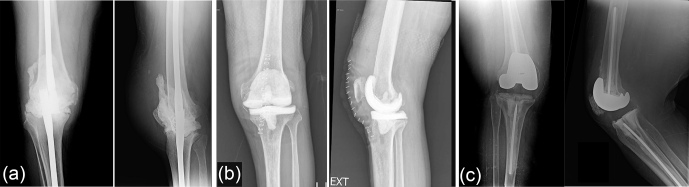
Types of antibiotic knee spacer: **(a)** static cement spacer, **(b)** articulating cement spacer, and **(c)** low-friction prosthetic spacer.

Total knee arthroplasty (TKA) is a highly effective treatment for advanced osteoarthritis and other debilitating conditions of the knee joint. Despite generally favorable outcomes, a prominent challenge associated with TKA is periprosthetic joint infection (PJI). PJI is the most common mode of failure for both primary and revision TKA (Bozic et al., 2010; Geary et al., 2020), and it produces significant morbidity, mortality, and healthcare costs (Mponponsuo et al., 2022). The gold standard for PJI management typically involves a two-stage revision, with a broad range of success reported: between 37.1 % and 100 % (Romano et al., 2012; Tigani et al., 2013; Stammers et al., 2015). Using this treatment strategy, the infected prosthesis is removed, and an antibiotic-loaded spacer is temporarily implanted to help achieve infection control before definitive reimplantation of new components. Currently, there is considerable variability in the composition, design, and antibiotic regimen of spacers used in clinical practice. Three main types commonly employed include static cement spacers, articulating cement spacers, and prosthetic low-friction antibiotic (PALF) spacers (Fig. 1). Each type offers distinct advantages and considerations in terms of mechanical stability, antibiotic delivery kinetics, and ability to preserve joint function during the interstage period.

Despite widespread use in treating PJI, comparative studies evaluating the respective efficacy and outcomes for different spacer variants remain limited. Prior study has proposed that static spacers provide greater relief to infected and congested soft tissues, permitting better bacterial eradication (Faschingbauer et al., 2016). However, recent literature demonstrates that static spacers increase the risk of spacer displacement and bone loss between resection arthroplasty and revision (Shen et al., 2010). This may contribute to the instability and wound-healing problems associated with static spacers (Hofmann et al., 2005). In contrast, articulating cement spacers enable joint motion, protecting against scar formation and contracture (Thabe and Schill, 2007). The PALF spacer, a new or resterilized femoral implant articulating with a new polyethylene tibial insert, offers a lower coefficient of friction compared to cement articulating spacers (Lyons et al., 2019). In the cases of sterilized replanted spacers, research has suggested a superior interval and postoperative range of motion (ROM) compared with all-cement spacers (Fei et al., 2022).

Despite a small number of studies, no consensus in the literature has been achieved on an optimal spacer selection to maximize infection clearance. Moreover, it has been posited by infectious disease specialists that prosthetic spacers with an implant component may be inferior for pathogen eradication compared to antibiotic cement spacers. Comprehensive analyses that systematically compare infection eradication rates, functional outcomes, and complication rates among static, articulated, and PALF spacers are crucial to inform evidence-based decision-making and optimize treatment protocols. Contrasting the relative strengths and limitations of different antibiotic spacer types is crucial for both orthopedic surgeons and infectious disease specialists involved in PJI management. However, a gap in knowledge persists surrounding differential effectiveness between these spacer types. Thus, the purpose of this study was to compare outcomes following the initiation of two-stage exchange between patients with the placement of static cement, articulated cement, and PALF spacers for TKA PJI. We hypothesized that infection eradication, interstage period duration, functional outcomes, and adverse events would not significantly vary across these three groups.

## 2 Methods

This Institutional Review Board (IRB)-approved retrospective cohort study was conducted at an academic tertiary care center accredited by the Joint Commission International and the Accreditation Council of Graduate Medical Education – International. All study data were obtained via electronic medical record (EMR) information from multiple hospitals within a regional health system including academic facilities and community hospitals. EMR information was used to identify 320 patients who underwent two-stage revision for TKA PJI by any board-certified, fellowship-trained arthroplasty surgeon within the regional health system between January 2016 and December 2021. This endpoint was selected to ensure a minimum 2-year follow-up. All facilities across the regional health system employ a shared electronic health record, promoting consistent documentation continuity. Study data were collected from records by trained clinicians and recorded in an electronic database. Given the retrospective design, patient study consent was waived, as procedures were part of routine care.

All patients received antimicrobial therapy in conjunction with their surgical intervention based on microbiological culture data, when available. For all patients, infectious disease input was sought to help guide antimicrobial therapy, and antibiotic regimens followed the Infectious Diseases Society of America (IDSA) guidelines for PJI management (Osmon et al., 2013). The attending surgeon led the patient's diagnosis and course of surgical management. As the analysis of records was retrospective, specific details regarding operative technique, spacer antibiotic composition, and postoperative antibiotic treatment varied between subjects. The choice of antibiotic spacer type and formulation of antibiotic cement were subject to clinical discretion by the attending surgeon, assisted by consultation with infectious disease specialists and available culture data.

Inclusion criteria encompassed adult patients who received a first-time static cement, articulating cement, or prosthetic low-friction spacer for PJI following primary TKA. The varieties of articulating and prosthetic spacers included in this study are described in the Supplement (Table S1 in the Supplement). Adherence to the 2013 Musculoskeletal Infection Society (MSIS) criteria was confirmed for all patients to ensure consistency in defining PJI (Parvizi et al., 2013). Criteria for exclusion were as follows: a prior antibiotic spacer; failure to meet MSIS criteria; inadequate follow-up; debridement, antibiotics, and implant retention (DAIR) before spacer placement; or additional ipsilateral knee procedure(s) between index TKA and PJI. Data collected included age, sex, body mass index (BMI), smoking status, history of diabetes, rheumatoid arthritis, use of anticoagulant medication, and the American Society of Anesthesiologists (ASA) classification. The Charlson comorbidity index (CCMI) was calculated using each patient's medical comorbidities at the time of two-stage exchange. Results of synovial fluid culture and antibiotic-sensitivity testing, erythrocyte sedimentation rate (ESR) and C-reactive protein (CRP) levels at reimplantation, and explant procedure timing were collected. The type of antibiotic spacer was identified through review of postoperative radiographs and operative reports. The spacer antibiotic formulation was recorded from operative reports, where available.

### 2.1 Outcome measures

The primary outcome measure was failure prior to the 2-year follow-up, defined using the 2019 MSIS Outcome Reporting Tool (MSIS ORT) (Zielinski et al., 2024). Patients meeting the criteria for ORT Tier 1 or Tier 2 were considered a success; tiers 3 and 4 were considered failures. As spacer retention (Tier 3F) may not necessarily constitute failure in all cases, a separate sensitivity analysis was performed wherein patients who retained their spacer beyond 2 years were excluded from failures. If failure was observed, the date upon which failure was first documented was recorded. Secondary outcomes of interest included operative times for initial spacer insertion and reimplantation, completion of two-stage exchange at 1 year, discontinuation of suppressive antibiotics by 1 year following reimplantation, mortality, time to failure, duration of stay for initial PJI hospitalization, ROM and ambulatory status at the final follow-up, and occurrence of a significant adverse event (AE). Surgical time was defined by the duration from skin incision to closure. AEs included the following: delayed wound healing, bleeding or the need for transfusion, hematoma, contracture or knee stiffness, venous thromboembolism (VTE), decubital ulcer, fall, or another notable adverse event recorded in the chart. Readmissions at 30 and 90 d following the initial procedure were documented.

### 2.2 Statistical analysis

Continuous measures were summarized with the mean and standard deviation or median and interquartile range, depending on the distribution of the measure. Comparisons were made between the three groups using a one-way ANOVA F test or the Kruskal–Wallis test if the distribution departed from normality. Post hoc comparisons were made with the independent samples t test or the Wilcoxon rank sum test and were adjusted for multiplicity with the Benjamini–Hochberg procedure. Categorical variables were summarized using the frequency and percentages and compared between groups with the chi-square or Fisher exact test. There was no preplanned sample size or power analysis conducted for this study, as this was a retrospective observational cohort. The significance level was set at p=0.05 . All analyses were performed in SAS version 9.4 (SAS Institute Incorporated, Cary, NC).

## 3 Results

**Figure 2:**
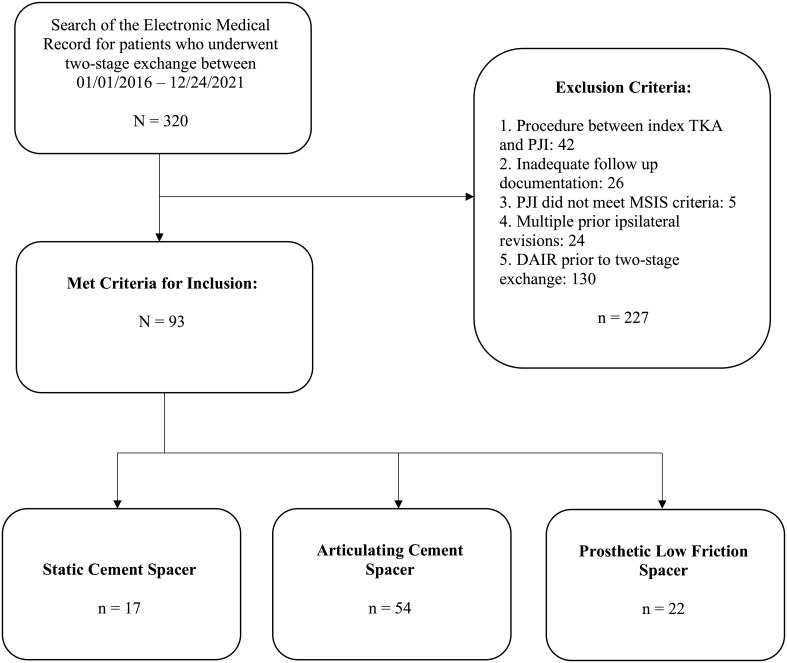
Consort diagram with patient inclusion, exclusion, and stratification.

Overall, 93 patients (50 female and 43 male) met the criteria for inclusion in this study. Types of spacers included static cement ( n=17 , 18.0 %), articulating cement ( n=54 , 57.4 %), and prosthetic low friction ( n=22 , 24.5 %) (Fig. 2).

No significant differences were observed between spacer groups with respect to age, sex, tobacco use, diabetes, rheumatoid arthritis, ASA or CCMI scores, or length of follow-up. The BMI in the static spacer group ( 39.8±12.2 ) was higher than that in the articulating cement group ( 32.6±5.4 ) and low-friction spacer group ( 32.0±7.1 ), p=0.002 ; however, significance was not maintained after controlling for multiple comparisons ( p=0.08 ) (Table 1).

**Table 1:** Demographic information by spacer type.

Demographic	Type of antibiotic spacer ( N=93 )			P value
characteristic	Static	Articulated	Prosthetic	
	cement	cement	low friction	
	( n=17 )	( n=54 )	( n=22 )	
Age, mean (SD)	61.4 (8.1)	63.8 (10.1)	66.0 (9.3)	0.34
Male sex, n (%)	8 (47)	27 (50)	8 (36)	0.56
BMI, mean (SD)	39.8 (12.2)	32.6 (5.4)	32.0 (7.1)	0.002^*^
Active tobacco use, n (%)	2 (11.8)	8 (14.8)	2 (9.1)	0.91
DM, n (%)	9 (53)	26 (48)	6 (27)	0.18
RA, n (%)	1 (6)	4 (8)	3 (14)	0.60
CCMI, mean (SD)	3.9 (2.0)	3.8 (1.7)	4.09 (1.4)	0.73
ASA, n (%)	–	–	–	0.99
II	3 (18)	10 (19)	4 (18)	–
III	13 (76)	38 (70)	15 (68)	–
IV	1 (6)	6 (11)	3 (14)	–
Follow-up (in days), mean (SD)	1552.4 (1040.4)	1721.4 (811.2)	1333.9 (455.9)	0.15

^*^ Significant difference in body mass index (BMI) did not remain after controlling for multiple comparisons ( p=0.08 ). Abbreviations: SD – standard deviation; DM – diabetes mellitus; RA – rheumatoid arthritis; CCMI – Charlson comorbidity index; ASA – American Society of Anesthesia score.

**Table 2:** Primary endpoint (failure) and spacer comparison.

	Static	Articulated	Prosthetic	P value
	cement	cement	low friction	
	( n=17 )	( n=54 )	( n=22 )	
Failure (MSIS tiers 3 or 4), n (%)	10 (58.8 %)	19 (35.2 %)	5 (22.7 %)	0.06
Failure category				0.15
Tier 3A/3C	1 (10.0)	3 (15.8)	0 (0.0)	
Tier 3B/3D	5 (50.0)	15 (79.0)	3 (60.0)	
Tier 3E	1 (10.0)	0 (0.0)	0 (0.0)	
Tier 3F	3 (30.0)	1 (5.2)	2 (30.0)	
Spacer antibiotic, n (%)				0.16
Gentamicin only	9 (52.9)	39 (72.2)	13 (59.1)	
Gentamicin and tobramycin	0 (0.0)	1 (1.85)	0 (0.0)	
Gentamicin and vancomycin	1 (5.9)	0 (0.0)	2 (9.1)	
Tobramycin only	0 (0.00)	1 (1.9)	1 (4.6)	
Tobramycin and vancomycin	4 (23.5)	4 (7.41)	2 (9.1)	
Gentamicin, tobramycin, and vancomycin	1 (5.9)	0 (0.0)	0 (0.0)	
Unknown	2 (11.8)	9 (16.67)	4 (18.2)	
Average duration of the interstage	113 (77)	91 (54)	128 (289)	0.05
period (in days), median (IQR)^1^				
ROM at the final follow-up, median (IQR)^2^	95 (45)	110 (30)	105 (40)	0.030^3^
Ambulatory status, n (%)	n=13	n=46	n=19	0.24
No assistance	2 (15.4)	9 (19.6)	9 (47.4)	–
Cane	2 (15.4)	14 (30.4)	4 (21.1)	
Walker	9 (69.2)	19 (41.3)	5 (26.3)	
Crutches	0 (0)	2 (4.4)	1 (5.3)	
Wheelchair	0 (0)	2 (4.4)	0 (0)	

^1^ Group sample sizes were 10 (static), 36 (articulating), and 16 (prosthetic low-friction) patients. ^2^ Group sample sizes included 14 (static) and 51 (articulating) patients. ^3^ The comparisons between the static cement spacer and the other two spacer types were initially significant (static versus articulated, p=0.01 ; static versus low friction, p=0.03 ); however, static versus low friction did not remain significant after controlling for multiple comparisons, whereas static versus articulated remained significant (static versus articulated, p=0.03 ; static versus low friction, p=0.06 ). Abbreviations: ROM – range of motion; IQR – interquartile range.

Failure, as defined by MSIS ORT, was observed in 10 patients (58.8 %) with a static cement spacer, 19 patients (35.2 %) with an articulating cement spacer, and 5 patients (22.7 %) with a prosthetic spacer, although this difference was not statistically significant ( p=0.06 ). Sensitivity analysis revealed that treating patients with spacer retention as successes did not significantly impact the failure rate comparison between groups (41.2 % versus 33.3 % 13.6 %, respectively; p=0.13 ). Most failures in each group were due to septic causes (Tier 3B or 3D), although the proportion did not differ significantly between spacer types. Antibiotics used in spacers included variable combinations of gentamicin, vancomycin, and tobramycin. While the inclusion of gentamicin was most commonly observed (66 patients), no difference in the overall frequency of antibiotic combinations were seen between groups ( p=0.16 ). Excluding patients who did not receive reimplantation, the median interstage durations were 113, 91, and 128 d, respectively ( p=0.05 ). Comparisons for median ROM at the final follow-up between static spacer and the other two spacer types were initially significant ( p=0.030 ). After controlling for multiple comparisons, static versus articulated spacer remained significant ( p=0.03 ), whereas static versus low friction and articulated versus low friction did not remain significant ( p=0.06 and 0.78, respectively). No significant differences were observed for the percentage of patients in each ambulatory status category at the final follow-up ( p=0.24 ) (Table 2).

**Table 3:** Comparison of PJI characteristics.

	Static	Articulated	Prosthetic	P value
	cement	cement	low friction	
	( n=17 )	( n=54 )	( n=22 )	
ESR (mm h^−1^) at time of	25.9 (15.9)	27.7 (18.6)	39.8 (27.8)	0.36
reimplantation, mean (SD)^1^				
CRP (mg dL^−1^) at time of	2.8 (3.3)	3.5 (7.4)	4.00 (6.7)	0.62
reimplantation, mean (SD)^1^				
Sinus tract, n (%)^2^	4 (23.5)	7 (13.5)	2 (9.1)	0.46
LOS (in days), mean (SD)^2^	8 (9)	5 (4)	5 (3)	0.13
Culture results, n (%)				0.58
Culture-negative	3 (17.7)	10 (18.5)	4 (18.2)	
Staphylococci	9 (52.9)	24 (44.0)	11 (50.0)	
*Staphylococcus aureus*	3 (17.7)	13 (24.1)	5 (22.7)	
*Staphylococcus lugdunensis*	1 (5.9)	3 (5.6)	1 (4.6)	
Coagulase-negative *Staphylococcus*	5 (29.4)	8 (14.8)	5 (22.7)	
*Streptococcus*	2 (11.8)	8 (14.8)	5 (22.7)	
Gram-negative	3 (18.0)	3 (5.6)	0 (0)	
Fungal^4^	0 (0.0)	1 (1.9)	0 (0)	
Other^5^	0 (0.0)	8 (14.8)	2 (9.09)	
MRSA	1 (5.9)	1 (1.9)	2 (9.1)	0.26
Multidrug-resistant pathogen, n %	5 (29.4)	12 (22.2)	8 (36.4)	0.44
Length of intravenous antibiotics	42 (5)	43 (3)	42 (3)	0.63
(in days), median (IQR)				
Length of oral antibiotics (in days),	49 (53)	183.5 (348)	181 (189)	0.33
median (IQR)^3^				

^1^ Group sample sizes were 9 (static), 38 (articulating), and 16 (prosthetic low-friction) patients. ^2^ Sample size was 52 patients for the articulating cement spacer group. ^3^ In total, 6 patients received oral antibiotics in the static group, 18  patients received oral antibiotics in the articulating group, and 17 patients received oral antibiotics in the prosthetic low-friction group. ^4^ The fungal species was *Aspergillus fumigatus*. ^5^ Other bacterial species included *Actinomyces* spp., *Anaerococcus octavius*, *Corynebacterium striatum*, *Dermabacter hominis*, *Enterococcus faecalis*, *Finegoldia magna*, and *Lactobacillus rhamnosus*. Abbreviations: SD – standard deviation; LOS – length of stay; MRSA – methicillin-resistant *Staphylococcus aureus*; IQR – interquartile range.

Values for ESR and CRP at the time of reimplantation were slightly elevated on average and did not differ significantly between groups ( p=0.36 and p=0.62 , respectively). The percentage of patients with a draining sinus tract at presentation and length of hospitalization for stage one were not significantly different between groups. Frequencies of infection with bacterial species were tabulated by category (Table 3), with no significant differences seen between spacer cohorts ( p=0.58 ). *Staphylococcus* spp., including *S. aureus*, *S. lugdunensis*, and other coagulase-negative *Staphylococci*, was the most frequently implicated pathogen. The frequencies of multidrug-resistant pathogens and methicillin-resistant *S. aureus* did not differ significantly between groups ( p=0.44 and p=0.26 , respectively). The median duration of intravenous antibiotic therapy was similar between all groups at 42 d ( p=0.63 ). The median duration of oral antibiotic therapy following reimplantation was 49±53 d for the static group, 184±348 d for the articulating group, and 181±189 d for the PALF group. However, this difference did not prove significant ( p=0.33 ).

Of patients who completed two-stage exchange, 41 % of the static spacer cohort, 35 % of the articulating cement cohort, and 21 % of the prosthetic low-friction cohort had received final components at 1 year, although differences did not prove significant ( p=0.43 ; Table 4). No difference in the mean operative duration was seen between groups during explantation ( p=0.86 ). However, the surgical duration for reimplantation was significantly longer in the static spacer group compared to the PALF spacer group (165.1 versus 124.8 min, p=0.006 ). In the prosthetic spacer group, 41 % of patients with adequate documentation had concluded all suppressive antibiotic therapy at 1 year; this is compared to 83 % in the static group and 67 % in the articulating group. However, only 41 patients (44.1 % of the sample) received oral antibiotic therapy, representing a limited fraction of the overall group. Overall mortality was low at 4 patients (4.3 %), with no significant differences seen between spacer types.

**Table 4:** Secondary endpoints.

	Static	Articulating	Prosthetic	P value
	cement	cement	low friction	
	( n=17 )	( n=54 )	( n=22 )	
Surgical time (minutes), mean (SD)				
Spacer insertion	118.4 (38.4)	113.9 (29.4)	113.3 (30.3)	0.86
Reimplantation	165.1 (52.6)	143.5 (33.7)	124.8 (30.0)	0.0079^3^
If second stage completed,	7 (41)	19 (35)	5 (23)	0.43
completed at 1 year, n (%)				
Off oral antibiotics at 1 year following	5 (83.3)	12 (66.7)	7 (41.2)	0.16
reimplantation, n (%)^1^				
Mortality, n (%)	2 (11.8)	2 (3.7)	0 (0)	0.19
Time to failure (in days), mean (SD)	148 (276)	243 (845)	391 (322)	0.36
Readmissions, n (%)^2^				0.52
None	6 (50)	29 (54)	14 (77.8)	
30 d	4 (33.3)	6 (11)	3 (16.7)	
90 d	2 (16.7)	4 (7)	1 (5.6)	
Experienced an AE, n (%)	9 (52.9)	12 (22.2)	4 (18.2)	0.030^4^
Delayed wound healing, n (%)	1 (5.9)	2 (3.7)	1 (4.6)	0.81
Bleeding/transfusion, n (%)	2 (11.8)	1 (1.9)	0 (0)	0.13
Hematoma, n (%)	2 (11.8)	0 (0)	0 (0)	0.030^5^
Contracture/stiffness, n (%)	0 (0)	3 (5.6)	0 (0)	0.76
VTE, n (%)	0	0	0	n/a
Decubital ulcer, n (%)	0	0	0	n/a
Fall, n (%)	2 (11.8)	3 (5.6)	1 (4.6)	0.72
Other significant AE, n (%)	5 (29.4)	5 (9.3)	1 (4.6)	0.06

^1^ Sample size was 6 (static), 18 (articulating), and 17 (prosthetic low-friction) patients. ^2^ Sample size was 12 (static), 41 (articulating), and 18 (prosthetic low-friction) patients. ^3^ Reimplantation time was significantly greater for static spacer compared to prosthetic low-friction spacer patients ( p=0.006 ). ^4^ The difference in the number of patients who experienced an adverse event between static cement spacer and other groups was significant even after adjustment (static versus articulated, p=0.03 ; static versus low friction, p=0.04 ). ^5^ While global testing was significant, post hoc pairwise tests showed no significant difference between groups. Abbreviations: SD – standard deviation; AE – adverse event; n/a – not applicable.

The total number of patients who experienced a perioperative or postoperative AE amounted to 9 (53 %) in the static cement group, 12 (22 %) in the articulating spacer group, and 4 (18 %) in the prosthetic low-friction group ( p=0.030 ), with the static group experiencing more AEs than both the articulated ( p=0.02 ) and the low-friction groups ( p=0.02 ). No difference was detected between the articulated and low-friction group ( p=0.77 ). However, after controlling for multiple post hoc comparisons, these two comparisons did not remain significant ( p=0.05 for both comparisons). Across the entire population, the most frequent AEs were falls (6 patients, 6.5 %) and delayed wound healing (4 patients, 4.3 %). In the static group, 2 patients developed a wound hematoma, compared to 1 in the prosthetic group and 0 in the articulating group ( p=0.03 ); however, post hoc pairwise testing did not demonstrate any significant differences between groups. Other notable adverse events included aseptic loosening (1), myocardial infarction (2), postoperative delirium (1), right-hand lower-extremity cellulitis (1), persistent knee drainage (1), knee arthrodesis (3), complex regional pain syndrome (1), and hypersensitivity dermatitis attributed to antibiotic therapy (1). Five patients experienced two or more AEs.

## 4 Discussion

Coupled with surging demand for TKA (Kurtz et al., 2007), the incidence of PJI results in significant clinical challenges and a sizable economic burden. The magnitude of this problem underscores the need for efficacious and reliable management, such as antibiotic spacer insertion. While two-stage revision with an antibiotic spacer is considered the gold standard for PJI treatment, its clinical course is not without obstacles and complications (Cochran et al., 2016). Several options for antibiotic spacer placement are available, although their comparative efficacy requires further investigation. This study aimed to provide insights into the similarities, advantages, and limitations of three commonly implemented spacer types.

The primary goal of this study was to compare failure between static cement, articulating cement, and prosthetic spacers. In discussion with infectious disease colleagues at our institution, questions have been raised about the potential inferiority of prosthetic low-friction spacers. It had been speculated that the implant component of the spacer may interfere with source control and offer a nidus for bacterial persistence. Here, no evidence of significantly greater effectiveness was observed for any singular spacer type. The overall failure rate for two-stage exchange in this population was 37 %, comparable to moderate rates reported in prior literature (Pangaud et al., 2019; Engesaeter et al., 2011). Between cohorts, failure rates ranged from 23 % in the PALF group to 59 % in the static spacer group. While statistical significance was not observed, this discrepancy is worth noting. Given relatively small group sizes for both static and prosthetic spacers, power may have limited our ability to detect an underlying prognostic difference. However, the equivalent frequency of septic revision between cohorts strengthens the argument for similar utility in infection clearance. Further studies with larger cohorts and possibly multicenter populations are warranted to definitively evaluate how efficacy may differ between spacers.

To our knowledge, this is the first study directly comparing PJI eradication across all three spacer variants, although comparisons have been made between individual types in prior studies. In one review examining 30 retrospective studies and 821 knees, Yu et al. (2019) found comparable success rates between all-cement articulating spacers and those containing prosthetic bio-inert materials, concurring with our results. Warwick et al. (2023) retrospectively compared static and articulating spacers, reporting significantly greater failure rates for static varieties. Our data regarding static spacers contrast with these findings and may represent new evidence of similar infection eradication using this type. Furthermore, the comparable failure rates for PALF spacers suggest that prosthetics with implanted components can be successfully deployed as antibiotic spacers.

The duration of intravenous (IV) and oral antibiotic therapy following two-stage exchange is a crucial management consideration. The IDSA and the International Consensus Meeting (ICM) on PJI currently recommend 4 to 6 weeks of IV interstage antibiotics (Osmon et al., 2013; Nagra et al., 2016; Gehrke et al., 2015). A key question addressed by our study was whether the required duration of antibiotic therapy varied significantly between spacer types, which could potentially suggest differences in effectiveness. Here, the median duration of IV antibiotics approximated this 6-week guideline for all groups. While no significant group differences were seen in the duration of suppressive oral antibiotics, it is worth noting that the static cement cohort approximated 7 weeks of therapy. To compare, both the articulating cement and prosthetic spacer groups showed a duration of approximately 6 months. However, the decision to prescribe oral therapy relies heavily on clinical discretion. As such, not all patients in the study received suppressive antibiotics, further decreasing power. Therefore, it is difficult to draw conclusions on observed differences in oral antibiotic duration, as they are likely due to small sample sizes. Other authors have evaluated extended suppressive therapy in the setting of debridement, antibiotics, and implant retention (DAIR) for TKA PJI, finding significantly lower failure rates up to 12 months with suppressive therapy (Chao et al., 2024; Shah et al., 2020). Future prospective studies with standardized IV and oral antibiotic regimens are needed to accurately assess how antimicrobial therapy may modulate failure in conjunction with spacer type.

Causal microbiology is an important consideration that may affect PJI outcomes. Although no significant differences between groups for culture results were observed in this study, *Staphylococcus* spp. was most frequently implicated across all groups and represented approximately half of all included PJI cases. Culture-negative, streptococcal, polymicrobial, and multidrug-resistant etiologies were also similarly distributed. Lee et al. (2010) and Akgun et al. (2017) have implicated *Staphylococcus* and *Streptococcus* spp. as risk factors for treatment failure, while others have reported additional risk with respect to fungal and mycobacterial etiologies (Lee et al., 2024). It is conceivable that the proportionate burden of high-risk pathogens such as *Staphylococcus aureus*, antibiotic-resistant species, and methicillin-resistant *Staphylococcus aureus* (Cheung et al., 2021) across groups may have contributed toward congruence in failure rates. Further study is needed to determine if separate spacer types demonstrate differential effectiveness against pathogens with varying virulence characteristics. Additionally, future studies may investigate the interplay between antibiotic selection, spacer type and composition, and causal microbiology.

Although our findings did not provide evidence of differential failure between spacer types, other potential complications and morbidity associated with spacer placement require separate appraisal. Previously, static spacers have been linked to prolonged immobilization, stiffness, reduced ROM, exposure difficulties, and decreased patient satisfaction (Park et al., 2010; Hofmann et al., 2005). Furthermore, static spacers are associated with higher complication rates, including falls, hematomas, contractures, and delayed wound healing. The heightened risk of complication may be partially attributable to frequent use in frailer patient populations, as this technique has been thought to enable faster removal and reimplantation (Park et al., 2010). We assessed group demographics to account for potential bias, finding no differences in age, comorbidities, or baseline surgical risk. Nevertheless, static spacers demonstrated a higher complication rate. Of note, our findings for operative duration contradict the notion that a static spacer truly facilitates expedient removal. Another recent study comparing static and articulating spacers reported similar results (Warwick et al., 2023). Collectively, these data challenge the existing paradigm and provide evidence of disadvantageously slower removal with static spacers. Static spacers are often utilized in cases involving greater bone loss and poor soft-tissue coverage. This may bear on the likelihood of adverse events, as compromised soft-tissue coverage can lead to wound-healing issues and persistent drainage (Grant et al., 2024). These findings may support the use of articulating or prosthetic spacers to mitigate complication risk in patients with additional predisposing factors. However, other literature has reported similar complication rates for both types when compared to static spacers (Voleti et al., 2013), and the precise interconnection of these factors remains uncertain.

We also observed a longer median interstage period for a PALF spacer compared to an articulating cement spacer. Although this difference did not achieve statistical significance, this may reflect a clinically valuable disparity. Published studies on interstage durations have reported varying results, with most intervals ranging between 80 and 100 d (Dieckmann et al., 2019; Vasarhelyi et al., 2022; Winkler et al., 2019; Aali Rezaie et al., 2018; Choi et al., 2011; Puetzler et al., 2024). In a retrospective study by Choi et al. (2012), four patients with prosthetic spacer who did not undergo reimplantation endorsed satisfactory joint function alongside infection control, and this spacer essentially served as a definitive treatment. Another study reported that several patients treated with prosthetic spacers delayed reimplantation by more than a year, accredited to sufficient comfort (Cuckler, 2005). While no patients in our study were definitively treated with low-friction spacers, the notably longer interstage duration could suggest that patients achieved greater comfort and functionality with this type. However, this remains speculative. Clinical decisions on proceeding with stage two are multifactorial, and articulating spacers may truly confer a quicker path to final components. This is a critical area for future prospective study, which would permit comprehensive evaluation of all factors involved in determining reimplantation timing for each spacer type.

Our study has several limitations. This was a retrospective cohort study involving patients from one regional health system, carrying all limitations intrinsic to this study design. Our modest sample size of 93 patients may have limited our power, precluding definitive conclusions. Given the retrospective design, the type of antibiotic spacer used was subject to the surgeon's clinical discretion, and we cannot account for the individual factors that influenced the surgeon's decision between spacer types in each case. Moreover, as patients treated by multiple surgeons were included, surgical and antibiotic details varied between cases. Although all antibiotic use followed IDSA guidelines, the IV, oral, and spacer-eluted antibiotic used to treat infection may have varied between infection with the same pathogen (such as *Staphylococcus aureus*). More dedicated study is needed to fully illustrate the symbiotic relationship between antibiotic selection and spacer type. In addition, data were not available for all patients for every variable or outcome of interest. Future prospective studies involving larger, multicenter cohorts should be performed to provide more robust evidence to support these findings and address the potential for underpowered results.

## 5 Conclusions

Periprosthetic joint infection imposes an increasing burden on the healthcare system. The gold standard of two-stage revision has been well studied, although investigations comparing available spacer variants are limited. While all spacer types evaluated here showed comparable success in infection eradication, there is evidence to suggest that prosthetic low-friction spacers may have a superior functional status and lower complication rates. Static spacers have demonstrated a higher adverse-event rate compared with articulating and prosthetic comparators, although this may be attributable to use in cases of diminished bone stock and poor soft-tissue support. Further study, including prospective randomized evaluation, is needed in the future.

## Supplement

10.5194/jbji-10-243-2025-supplement The supplement related to this article is available online at https://doi.org/10.5194/jbji-10-243-2025-supplement.

## Supplement

10.5194/jbji-10-243-2025-supplement
10.5194/jbji-10-243-2025-supplement The supplement related to this article is available online at https://doi.org/10.5194/jbji-10-243-2025-supplement.

## Data Availability

No publicly available data sets were used in this article. All patient data were collected and stored securely within institutional database software.

## Appendix

The supplement related to this article is available online at https://doi.org/10.5194/jbji-10-243-2025-supplement.

## References

[bib1.bib1] Aali Rezaie A, Goswami K, Shohat N, Tokarski AT, White AE, Parvizi J (2018). Time to Reimplantation: Waiting Longer Confers No Added Benefit. J Arthroplast.

[bib1.bib2] Akgun D, Trampuz A, Perka C, Renz N (2017). High failure rates in treatment of streptococcal periprosthetic joint infection: results from a seven-year retrospective cohort study. Bone Joint J.

[bib1.bib3] Bozic KJ, Kurtz SM, Lau E, Ong K, Chiu V, Vail TP, Rubash HE, Berry DJ (2010). The epidemiology of revision total knee arthroplasty in the United States. Clin Orthop Relat Res.

[bib1.bib4] Chao R, Rothenberger SD, Frear AJ, Hamlin BR, Klatt BA, Shah NB, Urish KL (2024). Benefits and Adverse Events Associated With Extended Antibiotic Use for One Year Following Periprosthetic Joint Infection in Total Knee Arthroplasty: A Prospective Cohort Analysis. J Arthroplast.

[bib1.bib5] Cheung GYC, Bae JS, Otto M (2021). Pathogenicity and virulence of Staphylococcus aureus. Virulence.

[bib1.bib6] Choi HR, von Knoch F, Zurakowski D, Nelson SB, Malchau H (2011). Can implant retention be recommended for treatment of infected TKA?. Clin Orthop Relat Res.

[bib1.bib7] Choi HR, Malchau H, Bedair H (2012). Are prosthetic spacers safe to use in 2-stage treatment for infected total knee arthroplasty?. J Arthroplast.

[bib1.bib8] Cochran AR, Ong KL, Lau E, Mont MA, Malkani AL (2016). Risk of Reinfection After Treatment of Infected Total Knee Arthroplasty. J Arthroplast.

[bib1.bib9] Cuckler JM (2005). The infected total knee: management options. J Arthroplast.

[bib1.bib10] Dieckmann R, Schmidt-Braekling T, Gosheger G, Theil C, Hardes J, Moellenbeck B (2019). Two stage revision with a proximal femur replacement. BMC Musculoskelet Disord.

[bib1.bib11] Engesaeter LB, Dale H, Schrama JC, Hallan G, Lie SA (2011). Surgical procedures in the treatment of 784 infected THAs reported to the Norwegian Arthroplasty Register. Acta Orthop.

[bib1.bib12] Faschingbauer M, Bieger R, Reichel H, Weiner C, Kappe T (2016). Complications associated with 133 static, antibiotic-laden spacers after TKA. Knee Surg Sports Traumatol Arthrosc.

[bib1.bib13] Fei Z, Zhang Z, Wang Y, Zhang H, Xiang S (2022). Comparing the Efficacy of Articulating Spacers in Two-Stage Revision for Periprosthetic Joint Infection Following Total Knee Arthroplasty: All-Cement Spacers vs Sterilized Replanted Metal-Polyethylene Spacers. Int J Gen Med.

[bib1.bib14] Geary MB, Macknet DM, Ransone MP, Odum SD, Springer BD (2020). Why Do Revision Total Knee Arthroplasties Fail? A Single-Center Review of 1632 Revision Total Knees Comparing Historic and Modern Cohorts. J Arthroplast.

[bib1.bib15] Gehrke T, Alijanipour P, Parvizi J (2015). The management of an infected total knee arthroplasty. Bone Joint J.

[bib1.bib16] Grant C, Chang J, Poehlein E, Green CL, Seidelman J, Jiranek W (2024). Static Versus Articulating Spacer: Does Infectious Pathogen Type Affect Treatment Success?. Clin Orthop Relat Res.

[bib1.bib17] Hofmann AA, Goldberg T, Tanner AM, Kurtin SM (2005). Treatment of infected total knee arthroplasty using an articulating spacer: 2- to 12-year experience. Clin Orthop Relat Res.

[bib1.bib18] Kurtz S, Ong K, Lau E, Mowat F, Halpern M (2007). Projections of primary and revision hip and knee arthroplasty in the United States from 2005 to 2030. J Bone Joint Surg Am.

[bib1.bib19] Lee J, Kang CI, Lee JH, Joung M, Moon S, Wi YM, Chung DR, Ha CW, Song JH, Peck KR (2010). Risk factors for treatment failure in patients with prosthetic joint infections. J Hosp Infect.

[bib1.bib20] Lee WS, Park KK, Cho BW, Park JY, Kim I, Kwon HM (2024). Risk factors for early septic failure after two-stage exchange total knee arthroplasty for treatment of periprosthetic joint infection. J Orthop Traumatol.

[bib1.bib21] Lyons S, Downes K, Habeck J, Whitham Z, Werger M, Stanat S (2019). Early to midterm results of “low-friction” articulating antibiotic spacers for septic total knee arthroplasty. Arthroplast Today.

[bib1.bib22] Mponponsuo K, Leal J, Puloski S, Chew D, Chavda S, Au F, Rennert-May E (2022). Economic burden of surgical management of surgical site infections following hip and knee replacements in Calgary, Alberta, Canada. Infect Control Hosp Epidemiol.

[bib1.bib23] Nagra NS, Hamilton TW, Ganatra S, Murray DW, Pandit H (2016). One-stage versus two-stage exchange arthroplasty for infected total knee arthroplasty: a systematic review. Knee Surg Sports Traumatol Arthrosc.

[bib1.bib24] Osmon DR, Berbari EF, Berendt AR, Lew D, Zimmerli W, Steckelberg JM, Rao N, Hanssen A, Wilson WR (2013). Diagnosis and management of prosthetic joint infection: clinical practice guidelines by the Infectious Diseases Society of America. Clin Infect Dis.

[bib1.bib25] Pangaud C, Ollivier M, Argenson JN (2019). Outcome of single-stage versus two-stage exchange for revision knee arthroplasty for chronic periprosthetic infection. EFORT Open Rev.

[bib1.bib26] Park SJ, Song EK, Seon JK, Yoon TR, Park GH (2010). Comparison of static and mobile antibiotic-impregnated cement spacers for the treatment of infected total knee arthroplasty. Int Orthop.

[bib1.bib27] Parvizi J, Gehrke T, Chen AF (2013). Proceedings of the International Consensus on Periprosthetic Joint Infection. Bone Joint J.

[bib1.bib28] Puetzler J, Hofschneider M, Gosheger G, Theil C, Schulze M, Schwarze J, Koch R, Moellenbeck B (2024). Evaluation of time to reimplantation as a risk factor in two-stage revision with static spacers for periprosthetic knee joint infection. J Orthop Traumatol.

[bib1.bib29] Romano CL, Gala L, Logoluso N, Romano D, Drago L (2012). Two-stage revision of septic knee prosthesis with articulating knee spacers yields better infection eradication rate than one-stage or two-stage revision with static spacers. Knee Surg Sports Traumatol Arthrosc.

[bib1.bib30] Shah NB, Hersh BL, Kreger A, Sayeed A, Bullock AG, Rothenberger SD, Klatt B, Hamlin B, Urish KL (2020). Benefits and Adverse Events Associated With Extended Antibiotic Use in Total Knee Arthroplasty Periprosthetic Joint Infection. Clin Infect Dis.

[bib1.bib31] Shen H, Zhang X, Jiang Y, Wang Q, Chen Y, Wang Q, Shao J (2010). Intraoperatively-made cement-on-cement antibiotic-loaded articulating spacer for infected total knee arthroplasty. Knee.

[bib1.bib32] Stammers J, Kahane S, Ranawat V, Miles J, Pollock R, Carrington RW, Briggs T, Skinner JA (2015). Outcomes of infected revision knee arthroplasty managed by two-stage revision in a tertiary referral centre. Knee.

[bib1.bib33] Thabe H, Schill S (2007). Two-stage reimplantation with an application spacer and combined with delivery of antibiotics in the management of prosthetic joint infection. Oper Orthop Traumatol.

[bib1.bib34] Tigani D, Trisolino G, Fosco M, Ben Ayad R, Costigliola P (2013). Two-stage reimplantation for periprosthetic knee infection: Influence of host health status and infecting microorganism. Knee.

[bib1.bib35] Vasarhelyi E, Sidhu SP, Somerville L, Lanting B, Naudie D, Howard J (2022). Static vs Articulating Spacers for Two-Stage Revision Total Knee Arthroplasty: Minimum Five-Year Review. Arthroplast Today.

[bib1.bib36] Voleti PB, Baldwin KD, Lee GC (2013). Use of static or articulating spacers for infection following total knee arthroplasty: a systematic literature review. J Bone Joint Surg Am.

[bib1.bib37] Warwick HS, Tan TL, Weiser L, Shau DN, Barry JJ, Hansen EN (2023). Comparison of Static and Articulating Spacers After Periprosthetic Joint Infection. J Am Acad Orthop Surg Glob Res Rev.

[bib1.bib38] Winkler T, Stuhlert MGW, Lieb E, Muller M, von Roth P, Preininger B, Trampuz A, Perka CF (2019). Outcome of short versus long interval in two-stage exchange for periprosthetic joint infection: a prospective cohort study. Arch Orthop Trauma Surg.

[bib1.bib39] Yu Q, Luo M, Wu S, Lai A, Sun Y, Hu Q, He Y, Tian J (2019). Comparison of infection eradication rate of using articulating spacers containing bio-inert materials versus all-cement articulating spacers in revision of infected TKA: a systematic review and meta-analysis. Arch Orthop Trauma Surg.

[bib1.bib40] Zielinski MR, Ziemba-Davis M, Meneghini RM (2024). Comparison of Delphi Consensus Criteria and Musculoskeletal Infection Society Outcome Reporting Tool Definitions of Successful Surgical Treatment of Periprosthetic Knee Infection. J Arthroplast.

