# Supplementary material for: No difference in failure between static, articulating, and prosthetic low-friction spacers for periprosthetic joint infection of total knee arthroplasty
Source: J Bone Jt Infect. 2025 Jul 30;10(4):243–53. doi: 10.5194/jbji-10-243-2025 (PMC12311390; doi:10.5194/jbji-10-243-2025)
Supplement: The supplement related to this article is available online at https://doi.org/10.5194/jbji-10-243-2025-supplement. [file jbji-10-243-2025-supplement.pdf]

Supplement of J. Bone Joint Infect., 10, 243–253, 2025
https://doi.org/10.5194/jbji-10-243-2025-supplement
© Author(s) 2025. CC BY 4.0 License.

JBJI
Journal of Bone and Joint Infection
Open Access

Supplement of

No difference in failure between static, articulating, and prosthetic low-friction spacers for periprosthetic joint infection of total knee arthroplasty

Michael F. Shannon et al.

Correspondence to: Kenneth L. Urish (ken.urish@pitt.edu)

The copyright of individual parts of the supplement might differ from the article licence.

Supplementary Material

Table S1: Types of Articulating Cement and Prosthetic Low-Friction Spacers

Spacer Type	Brand and Manufacturer
**Articulating Cement Spacer**	• Simplex® HV, Stryker (Kalamazoo, Michigan, USA)<br>• SpinePlex®, Stryker (Kalamazoo, Michigan, USA)<br>• Triathlon®, Stryker (Kalamazoo, Michigan, USA)<br>• P.F.C. SIGMA®, Depuy Synthes (Raynham, Massachusetts, USA)<br>• StageOne™, Zimmer Biomet (Warsaw, Indiana, USA)<br>• REMEDY®, Osteoremedies (Memphis, Tennessee, USA).<br>• InterSpace®, Exactech (Gainesville, Florida, USA)<br>• Refobacin®, Zimmer Biomet (Warsaw, Indiana, USA)<br>• Cobalt® MVG, Enovis (Wilmington, Delaware, USA).<br>• SMARTSET™, Depuy Synthes (Raynham, Massachusetts, USA)
**Prosthetic Low-Friction Spacer**	• P.F.C. SIGMA®, Depuy Synthes (Raynham, Massachusetts, USA)<br>• Triathlon®, Stryker (Kalamazoo, Michigan, USA)<br>• Vivacit-E®, Zimmer Biomet (Warsaw, Indiana, USA)<br>• GENESIS II, Smith & Nephew (London, UK).<br>• LEGION, Smith & Nephew (London, UK).
